# Critical appraisal of evidence supporting prescription of psychedelics from clinic websites in Ontario, Canada

**DOI:** 10.1371/journal.pone.0309911

**Published:** 2024-10-24

**Authors:** Kyurim Kim, Abban Yusuf, Abhimanyu Sud, Nav Persaud, Abirami Kirubarajan, Monique Moller, Taryn Lloyd, Braden O’Neill

**Affiliations:** 1 Temerty Faculty of Medicine, Undergraduate Medical Education, University of Toronto, Toronto, ON, Canada; 2 MAP Centre for Urban Health Solutions, St. Michael’s Hospital, Unity Health Toronto, Toronto, ON, Canada; 3 Primary Care and Population Health Systems, Humber River Hospital, North York, ON, Canada; 4 Temerty Faculty of Medicine, Department of Family and Community Medicine, University of Toronto, Toronto, ON, Canada; 5 Postgraduate Medical Education, McMaster University, Hamilton, ON, Canada; 6 Centre for Addiction and Mental Health, Toronto, ON, Canada; 7 Department of Emergency Medicine, St. Michael’s Hospital, Unity Health Toronto, Toronto, ON, Canada; University of Brescia: Universita degli Studi di Brescia, ITALY

## Abstract

Psychedelics, including ketamine, 3,4-Methyl enedioxy methamphetamine (MDMA), and psilocybin, have gained attention for their potential therapeutic role in mental health treatment. While recreational use is prohibited in Canada, medicinal exemptions can be granted. There are several psychedelic clinics in Ontario, Canada, promoting the use of psychedelics for a variety of medical indications. Our objective was to identify the indications for which psychedelics are being prescribed in Ontario clinics and assess the quality of evidence used to support these claims. Internet searches were conducted using Google and Bing to identify psychedelic clinics in Ontario. Inclusion criteria was as follow: clinics were physically located in Ontario, had a functioning website link, and demonstrated involvement of a licensed physician or nurse practitioner. Identified clinics were evaluated for their claims of effectiveness, the quality of evidence used to support these claims, and statements on psychedelic-related harms. The cited studies were appraised for quality using Oxford Centre for Evidence-Based Medicine Levels of Evidence, “level 5” being the lowest quality and “level 1” being the highest quality. Out of 200 search results, 10 psychedelic clinic websites met our inclusion criteria. These clinics advertised psychedelics for 47 medical conditions, most commonly for depression. Only 2 out of 10 clinics described potential risks associated with psychedelic use. There were 29 studies cited by these websites, majority coming from “level 4” evidence consisting of case-series and case-control studies. Overall, the cited evidence quality was low to moderate. Psychedelic clinics in Ontario promote a wide range of medical indications for psychedelics using primarily low to moderate “level 4” evidence. There is limited information shared on the potential adverse effects of psychedelics. Our study emphasizes the importance of using transparent and high-quality evidence by clinics and clinicians to ensure safe and effective use of psychedelics in mental health treatments.

## Introduction

Psychedelics are a class of compounds known for their psychoactive effects. They include substances such as ketamine, 3,4-Methyl enedioxy methamphetamine (MDMA), and psilocybin, and have been explored for potential therapeutic applications in mental health treatments. Ketamine is a dissociative drug that alters sensations and interrupts the pain signaling pathway in the brain, mainly through its role as an N-methyl-D-aspartate (NMDA) receptor antagonist [1]. It is commonly prescribed and administered by health care professionals in hospital settings for anesthesia and acute pain management [2]. MDMA stimulates serotonin and dopamine pathways and can cause positive short-term effects on mood. Unlike ketamine, which is commonly used in medicine, MDMA has no currently approved therapeutic indications and is an illegal substance in most jurisdictions [3]. It is primarily used for recreational purposes. Psilocybin is one of the active ingredients in “magic mushrooms’’ and acts as a hallucinogen, altering emotions and senses. Psilocybin is also used recreationally for its psychological effects [4]. Some individuals describe consuming these psychedelics routinely in very low doses, known as microdosing, for perceived mental health benefits such as improvement in mood and anxiety [5].

In Canada, these three substances are regulated under the *Controlled Drugs and Substances Act*, under which unauthorized production, sale, or possession is illegal [2–4]. Although recreational psychedelic use in Canada is illegal under the *Controlled Drugs and Substances Act*, exemptions for use can be granted by Health Canada for medicinal or scientific purposes. In recent years, psychedelics have gained popularity as interventions for mental illnesses such as depression, post-traumatic stress disorder, and end-of-life suffering [6]. A 2021 CBC news article reported patients’ experiences regarding how psychedelic-assisted therapy, a therapeutic approach involving the use of psychedelics in conjunction with psychotherapy under the supervision of a licensed professional, dramatically improved their quality of life [7]. Despite the regulations set by the *Controlled Drugs and Substances Act*, stores selling psychedelic substances have opened around Ontario, allowing customers to walk-in and purchase psilocybin without a prescription [7]. While technically illegal under Canada’s current legislative framework, these shops remain open, with news reports describing that their inventories are periodically confiscated by police and that business resumes once the inventory is replenished [8,9]. With substantial attention on psychedelics and their potential therapeutic role, there has been a rapid increase in investments in the psychedelic market with a growth rate in the United States that is projected to exceed that of the cannabis market [10]. Financial investments in the psychedelic industry have taken off in late 2010s and early 2020s, and companies aim to commercialize therapeutic applications of psychedelics [11]. Efforts are made in many countries to gain full federal regulatory approval of psychedelic medicine, highlighting the growing interest in psychedelic-assisted therapy and the potential impact on future health policies [11]. With a surge in popularity and accessibility, it becomes important to evaluate the evidence behind the risks and efficacy.

Systematic reviews investigating the efficacy of ketamine, MDMA, and psilocybin for mental health disorders have demonstrated some effectiveness, particularly for certain treatment-resistant conditions [12–14]. One randomized control trial estimated that ketamine is comparable in efficacy to electroconvulsive therapy for people with treatment-resistant major depression; participants in this study received two 40-minute ketamine infusions per week, for 3 weeks [15]. A randomized placebo-controlled phase 3 trial showed MDMA-assisted therapy reduced PTSD symptoms and functional impairment in a diverse population and was well-tolerated [16]. In a double-blind, randomized, phase 3 clinical trial, MDMA was compared with placebo for the treatment of severe PTSD, including those with common comorbid mental health conditions [17]. The data indicated that MDMA-assisted integrative therapy sessions are highly efficacious in individuals with severe PTSD, reducing PTSD symptoms and functional impairment with high safety and tolerability [17]. However, given the relative infancy of structured clinical.

Research into these substances, more evidence is required to understand both short-term and long-term effects of psychedelics [6]. Ketamine and MDMA are addictive substances that can be prone to misuse, particularly if medicines are delivered in an unsafe and unsupervised setting. Repeated exposure has been shown in animal models to cause neurotoxicity in the central nervous system, although these studies generally use doses much greater than what is promoted for human administration [18,19]. A survey of American psychiatrists’ attitudes towards the use of psychedelics for therapeutic indications showed that despite recent literature highlighting therapeutic benefits of psychedelics, American psychiatrists appear apprehensive about the potential adverse effects associated with their use [20]. A modest majority expressed doubts regarding the suitability of psychedelics for mental health treatments, even in adjunction to psychotherapy [20]. There was an agreement among psychiatrists that further research is required to evaluate the therapeutic potential of psychedelics [20]. Considering the potential risks associated with these substances, it is essential to critically evaluate the information available.

According to one anonymous survey of 1221 people, 62% of the participants used internet websites as their main source of information about psychedelics, making internet webs sources the second most common source of information about psychedelics after personal experimentation [21]. Websites of clinics that promote psychedelics are an important source of information about these substances. To assess the information available on psychedelic clinic websites, we adapted a process used by previous comparable studies [22,23], and conducted a narrative review of the evidence used on the psychedelic prescribers’ websites to support the claims of psychedelic use for various therapeutic indications. Our objective was to identify the indications for which psychedelic medications are being prescribed in Ontario clinics and assess the quality of evidence used to support these claims.

## Methods

We conducted a narrative review of Ontario, Canada psychedelic clinic websites. We conducted internet searches between July 11–31, 2023, using Google and Bing, the two search engines with the greatest market share in North America to identify psychedelic clinics [24]. Searches were conducted using the keywords: *psychedelic*, *clinic*, and *Ontario*. These psychedelic clinics are privately owned, outpatient clinics in the community where anyone can independently book an appointment without a physician referral. In situations where a parent company owns or manages several psychedelic clinics, we first verified that a physical location was present in Ontario before analyzing research evidence cited on the parent website. Our searches were limited to the top 100 results each from Google and Bing (200 results in total), which is an established approach for online searches of this type [25]. Inclusion criteria involved clinics that were physically located within Ontario, had a functioning website link, and demonstrated presence of a prescriber licensed in Ontario: either a physician licensed with the College of Physicians and Surgeons of Ontario or a nurse practitioner who was licensed with the College of Nurses of Ontario, which serve as regulatory bodies for professional practice.

Two trained reviewers (KK and AY) worked in parallel to screen the top 100 results from each search engine against the inclusion criteria. Conflicting assessments were resolved by a third reviewer (BO). Afterwards, both reviewers independently examined all pages of included psychedelic clinic websites. We identified and documented any claims of effectiveness mentioned on the websites (e.g. “psychedelics help manage depression”) as well as the peer-reviewed supporting evidence cited for these claims. We limited our critical appraisal to peer-reviewed articles cited by these websites to focus on published research evidence used in support of these claims of effectiveness. The data and results were recorded in Google Sheets and analyzed using descriptive statistics.

All evidence collected was evaluated for its quality using the Oxford Centre for Evidence-Based Medicine (OCEBM) Levels of Evidence, an established framework used to assess the quality of evidence [26]. Under this framework, systematic reviews are initially rated as the highest level of evidence, “level 1”, with other studies ranked sequentially lower in quality. The lowest level of evidence, “level 5”, is reserved for studies conducted using animal disease models or mechanism-based reasoning. We classified studies using this approach and then ‘upgraded’ or ‘downgraded’ the level of evidence ascribed to each included study based on the OCEBM Levels of Evidence guidance (for example, where a study was described as a ‘systematic review’ but we assessed it to be poor quality, we downgraded this to a lower level, rather than Level 1 which is the ‘initial’ level for systematic reviews; we also did this for all other study types). We differentiated between reviews (non-appraisable) and systematic reviews (level 1) by analyzing the methods section of the studies. It was categorized as review if the study summarized and synthesized existing research on a particular topic without strict criteria for study selection. It was categorized as a systematic review if the study employed a clear predetermined criterion to search, select, appraise, and analyze relevant studies, with high replicability and reliability. There are other approaches available for appraising the quality of evidence used to make recommendations, such as the GRADE system, which is typically used to rate the quality of evidence in systematic reviews and evidence syntheses [27]. Since our focus was on assessing the quality of studies used on clinic websites to support their recommendations, we chose the OCEBM Levels of Evidence system, which we believe was most appropriately aligned with our objectives.

Two reviewers (KK and AY) independently assessed the quality of evidence, and any discrepancies were resolved via discussion with a third reviewer (BO). We reported the overall quality of evidence and the quality of evidence specific to each indication. Furthermore, our study included information on the total number of psychedelic clinics identified in Ontario, total number of claims, proportion of evidence-supported claims, statements on psychedelic-related harms, and any reported costs of therapy.

### Ethics statement

This study did not collect or analyze any data from human subjects, and no human subjects were enrolled. Ethical review and approval was not required, and no consent process was collected.

## Results

### Overview of cited evidence from clinic website

We characterized the quality of cited evidence by evaluating whether each study had a clear hypothesis, control group, power calculation, randomization, allocation concealment, intention to treat analysis, and blinding when applicable. Among the 29 studies that were referenced, the largest group was randomized controlled trials (RCTs) (7/29, 24%), followed by reviews (6/29, 21%) and systematic reviews (5/29, 17%) (Tables 1 and S1). For reviews and systematic reviews, criteria related to experimental design were not applicable as they synthesized existing research. The review studies were not graded as the Oxford Centre for Evidence-Based Medicine system does not include general reviews in their levels of evidence hierarchy. None of the systematic reviews had a clear hypothesis, but 3 had a clear objective, with explicit study selection criteria. The RCTs included a total of 447 participants, with the participant numbers ranging from 18 to 158 (median = 54, IQR = 26.5–82). 4 out of 7 (57%) studies had clearly stated hypotheses and all 7 (100%) had control groups and randomization. 4 studies (57%) completed a power calculation, and likewise, 4 (57%) employed an intent-to-treat analysis. All 7 (100%) RCTs had double blinding, while 4 (57%) trials used allocation concealment. Three RCTs (3/7, 43%) stated that blinding was difficult due to lack of ketamine effects in placebo groups, potentially resulting in biased patient reporting. Two (2/7, 29%) RCTs stated that if participants did not respond to double-blind or discontinued the study early due to lack of efficacy, they were administered open label standard ketamine dose. One RCT (1/7, 14%) stated that by the last infusion, 50% of ketamine cases guessed correctly about their assigned treatment.

**Table 1:** Distribution of types of studies cited by psychedelic-prescribing clinics.

Type of study
Randomized controlled trial (n = 7, 24%)
Review (n = 6, 21%)
Systematic review/ meta-analysis (n = 5, 17%)
Case series/report (n = 4, 14%)
Survey (n = 2, 7%)
Cross-sectional study (n = 2, 7%)
Expert opinion (n = 2, 7%)
Clinical trial (” noncontrolled” or nonrandomized) (n = 1, 3%)

There were 4 case series/ reports (14%), with a total of 195 participants and study sample sizes between 18 to 108 (median = 34.5, IQR = 19.5–64). None of the studies explicitly stated a hypothesis, and none conducted a power calculation. Only 1 out of 4 case series (25%) performed an intent-to-treat analysis. Criteria such as control groups or blinding were not applicable as they did not have comparison groups.

Out of 29 studies included in our analysis, 2 surveys (7%) were cited, with 321 and 886 participants respectively. They involved self-reports of the participants’ experience, at various time points before and after psychedelic use. One of the surveys (50%) had a clear hypothesis and neither of them (0%) completed a power calculation. Two out of 29 references (7%) were cross-sectional studies, with sample sizes of 235 and 9016. These cross-sectional studies involved observational data collection to assess associations among variables at a single point in time, and unlike surveys, did not include patient self-report. Neither of these studies had a clear hypothesis or a power calculation. Lastly, 2 editorials (7%) and 1 non-randomized clinical trial (3.5%) were cited by psychedelic clinic websites. The clinical trial study tested the safety and efficacy of repeated-dose IV ketamine for acute treatment of treatment resistant depression (TRD). The clinical trial involved 10 participants and performed an intent-to-treat analysis. The clinical trial study design did not include control group, power calculation, randomization, allocation concealment, nor blinding.

All analyzed studies enrolled adult participants who were 18 years or older; none included participants under 18 years of age. Of the 16 studies assessed that included human participants and reported sample sizes (n = 16), there were in total 11,110 participants (median = 61, IQR = 22–177).

### Results overview of identified clinic websites

**Fig 1:**
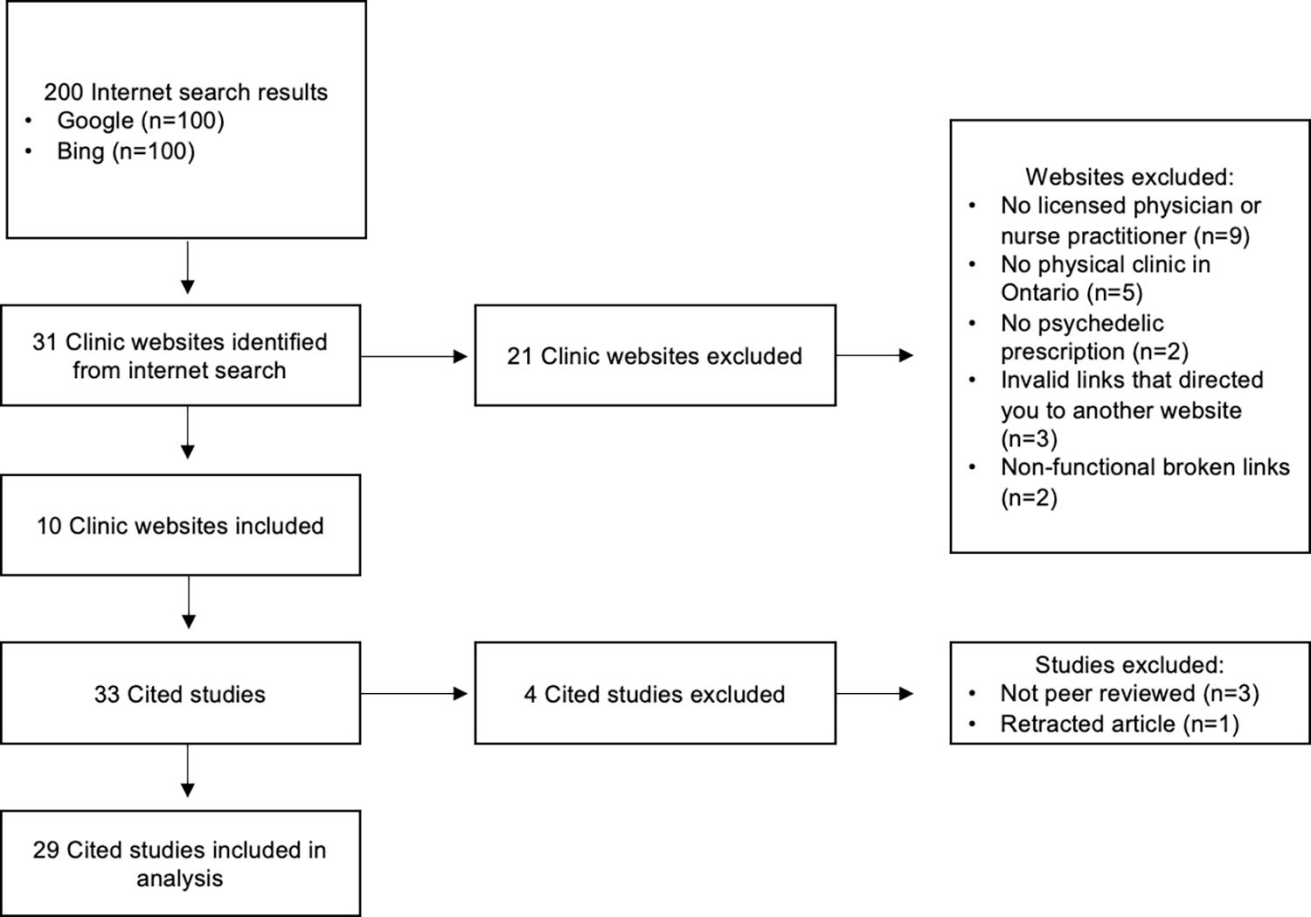
Study flow diagram.

Out of 200 search results, we identified a total of 31 potentially eligible psychedelic clinic websites in Ontario. 21 websites were excluded for following reasons: 9 websites (43%) did not have any licensed physician or nurse practitioner involvement; 5 websites (24%) did not have a physical clinic located in Ontario; 2 websites (9.5%) did not state that they prescribed psychedelics; 3 (14%) were invalid links that directed to another website; and 2 websites (9.5%) were non-functional links (Fig 1).

**Fig 2:**
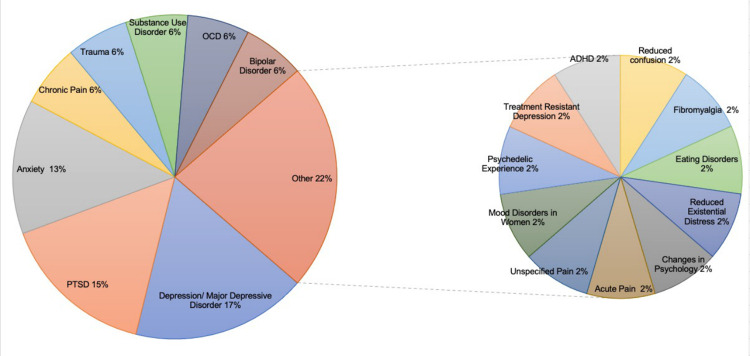
Medical indications claimed by psychedelic clinic websites.

Out of the 10 clinics that met our inclusion criteria, psychedelics were advertised for 47 unique medical conditions (Fig 2). The most frequently reported condition was depression or major depressive disorder (n = 8, 17%). Other indications include post-traumatic stress disorder (n = 7, 15%), anxiety (n = 6, 13%), chronic pain (n = 3, 6%), trauma (n = 3, 6%), substance use disorder (n = 3, 6%), obsessive compulsive disorder (n = 3, 6%), and bipolar disorder (n = 3, 6%). Only 2 out of 10 clinics (20%) described the potential risks and harms associated with psychedelic use. The potential side effects outlined on these websites ranged from milder symptoms such as nausea, elevated heart rate or blood pressure, and dizziness, to more severe symptoms such as cognitive dissociation, changes in liver or kidney function, or blood in urine. Nine out of 10 psychedelic clinics offered concurrent psychotherapy. The one clinic that did not offer psychotherapy promoted ketamine as an alternative physical pain treatment.

Here

33 different studies were referenced in these clinic’s websites to support their claims. 4 cited studies were not analyzed for their quality of evidence because 3 were non-peer reviewed sources such as internet websites or newsletters and 1 was retracted from publication (Fig 1). The remaining 29 studies investigated the outcome of psychedelics for a variety of indications (n = 48) such as depression/ major depressive disorder (n = 10, 21%), TRD (n = 10, 21%), post-traumatic stress disorder (n = 6, 13%), anxiety (n = 4, 8%), substance use disorder (n = 4, 8%), and bipolar disorder (n = 4, 8%) (Table 2).

**Table 2:** Medical indications for psychedelics from included studies and the types of study for each indication.

Indications for medical psychedelics (n = 48) and types of study for each indication (n = 29)
Depression/ Major depressive disorder (n = 10, 21%) • Systematic review (3/29, 10%) • RCT (2/29, 7%) • Case series (2/29, 7%) • Retrospective analysis (1/29, 3%) • Editorial (1/29, 3%) • Narrative review (1/29, 3%)
Treatment- resistant depression (n = 10, 21%) • Systematic review (3/29, 10%) • RCT (2/29, 7%) • Review (2/29, 7%) • Clinical trial (1/29, 3%) • Non-randomized trial (1/29, 3%) • Case series (1/29, 3%)
Post-traumatic stress disorder (PTSD) (n = 6, 13%) • RCT (2/29, 7%) • Review (2/29, 7%) • Systematic review (1/29, 3%) • Editorial (1/29, 3%)
Anxiety (n = 4, 8%) • Review (2/29, 7%) • Systematic review (1/29, 3%) • RCT (1/29, 3%)
Substance use disorder (n = 4, 8%) • Systematic review (2/29, 7%) • RCT (1/29, 3%) • Review (1/29, 3%)
Bipolar disorder (n = 4, 8%) • Systematic review (1/29, 3%) • Review (1/29, 3%) • Case series (1/29, 3%) • Editorial (1/29, 3%)
Mood disorders in women (n = 1, 2%) • Review (1/29, 3%)
Mood disorders (n = 1, 2%) • Review (1/29, 3%)
Trauma (n = 1, 2%) • Case series (1/29, 3%)
Eating disorder (n = 1, 2%) • Systematic review (1/29, 3%)
Suicidality (n = 1, 2%) • Review (1/29, 3%)
Changes in psychological insight (n = 1, 2%) • Survey (1/29, 3%)
Effects of psychedelics and cannabis combined (n = 1, 2%) • Survey (1/29, 3%)
Decreased “default mode network” activity (n = 1, 2%) • Review (1/29, 3%)
Obsessive compulsive disorder (OCD) (n = 1, 2%) • Systematic review (1/29, 3%)
Pain (unspecified) (n = 1, 2%) • Review (1/29, 3%)

### Evaluations of the quality of evidence

**Fig 3:**
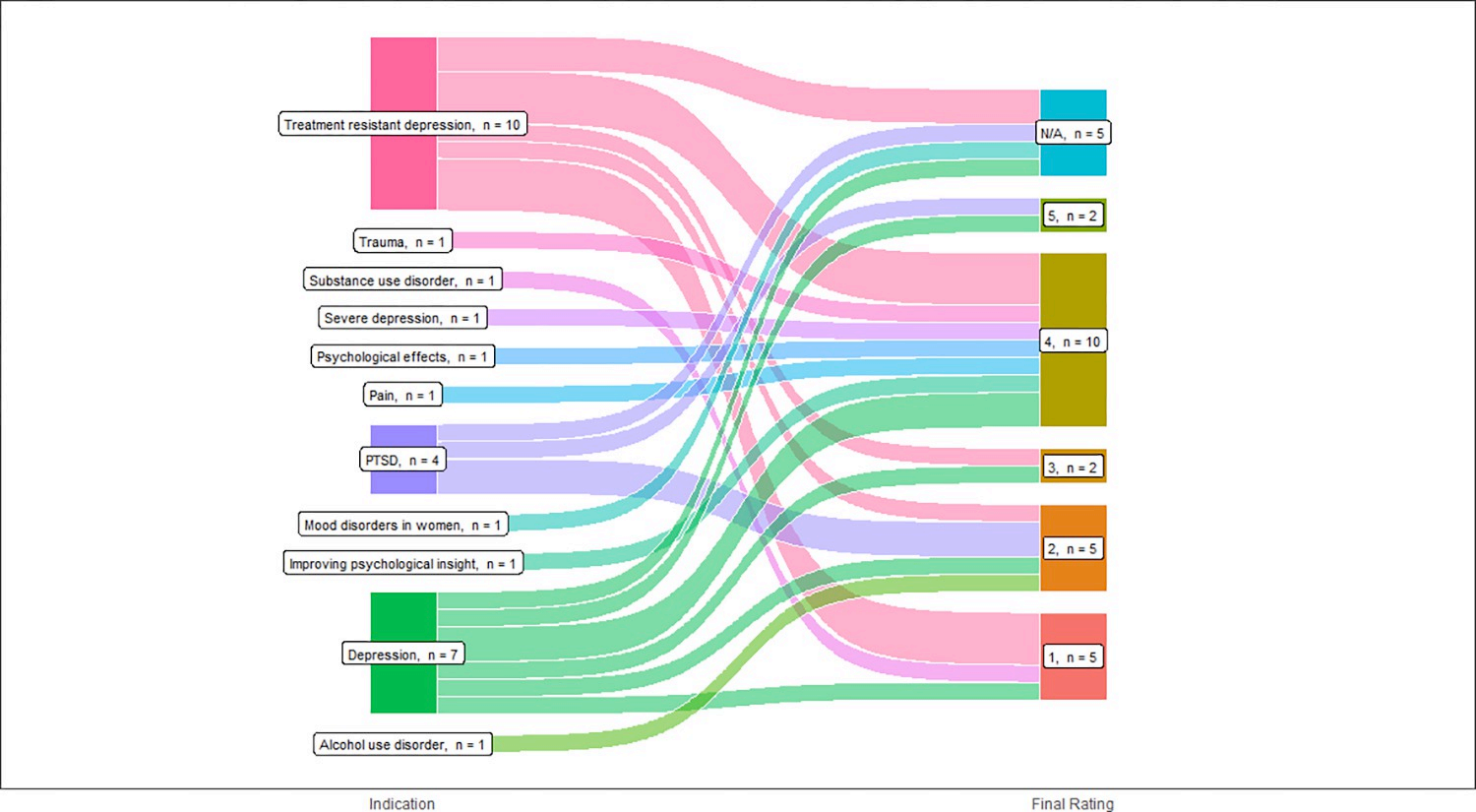
Level of evidence for medical indications.

**Fig 4:**
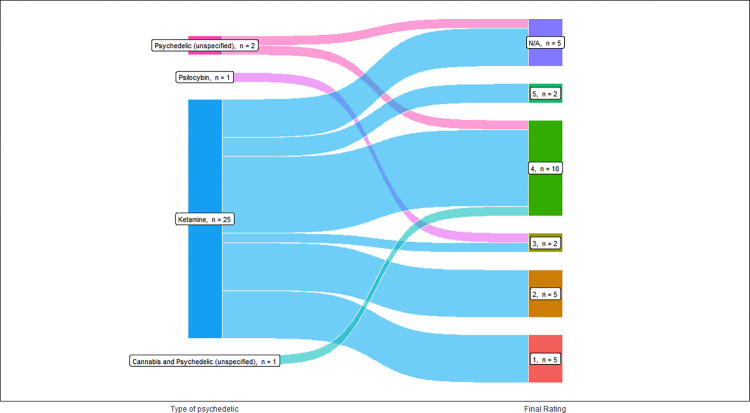
Level of evidence for type of psychedelic.

Among the 29 studies included in our analysis, the majority was lower quality “level 4” evidence (Table 3). The medical indications explored in the cited studies were matched to the 5 levels of evidence (Fig 3). Specific types of psychedelics were matched to its corresponding study level (Fig 4). Most included studies reported using ketamine and were level 4 studies.

**Table 3:** Quality of included cited studies.

Level of evidence
Level 1 (n = 5, 17%)
Level 2 (n = 5, 17%)
Level 3 (n = 2, 7%)
Level 4 (n = 10, 34%)
Level 5 (n = 2, 7%)
Unable to classify (reviews, basic science studies) (n = 5, 17%)

Level 1 (highest level of evidence): Systematic reviews.

Level 2: Randomized controlled trials.

Level 3: Nonrandomized, cross-sectional, and cohort studies.

Level 4: Case-series, case-control.

Level 5 (lowest level of evidence): Mechanism-based reasoning, expert opinions, descriptive studies.

### Psychedelic types and route of administration

Overall, 25 studies (86%) utilized ketamine alone; 1 study (3%) used psilocybin alone; 2 studies (7%) used a combination of psychedelics such as ketamine, psilocybin, 3,4-Methyl​enedioxy​methamphetamine (MDMA), and Lysergic acid diethylamide (LSD); and 1 study (3%) did not specify the type of psychedelic used (Table 4).

**Table 4:** Type of psychedelic from included cited studies.

Type of psychedelic
Ketamine (n = 25, 86%)
Psilocybin (n = 1, 3%)
Combination of psychedelics such as ketamine, psilocybin, MDMA, and LSD (n = 2, 7%)
Not specified (n = 1, 3%)

Regarding the route of administration, 14 studies (48%) examined intravenous infusions; 1 study (3%) examined intravenous injection; 1 study (3%) examined oral administration; 1 study (3%) examined sublingual administration; 1 study (3%) examined intranasal administration; 1 study (3%) examined sublingual and/or intramuscular administration; and 10 studies (35%) did not specify the route of administration used in their study (Table 5).

**Table 5:** Psychedelic route of administration from included cited studies.

Psychedelic route of administration
Intravenous infusion (n = 14, 48%)
Intravenous injection (n = 1, 3%)
Oral (n = 1, 3%)
Sublingual (n = 1, 3%)
Intranasal (n = 1, 3%)
Sublingual, intramuscular, or both (n = 1, 3%)
Not specified (n = 10, 35%)

### Costs

The costs of obtaining psychedelic medicines, most of which incorporated some ‘therapy’ such as psychotherapy in addition to the medicine itself, varied among the 10 clinics included in our analysis. Only the clinics that used ketamine for psychedelic therapy provided cost breakdowns. Five clinics (50%) did not provide a pricing estimate on their website; those that did charged anywhere between $200–1000 CAD per individual treatment. The clinics suggested that a patient may need anywhere from 4–8 treatments/sessions. Two clinics offered packages, starting at $5920 CAD for 6 sessions of psychedelic exploration or $9255 CAD for 2 sessions with a therapist in addition to 9 sessions of psychedelic exploration. No clinics specified the durability of therapeutic effect and how often these sessions are required. One clinic stated that the psychedelic-assisted therapy will have “long lasting” effects but did not specify further.

## Discussion

### Principal findings

Our narrative review of Ontario psychedelic clinic websites established that most evidence supporting psychedelic prescription is low to moderate quality. The largest proportion of evidence supporting the use of psychedelics in medical settings was from level 4, consisting of case-series or case-control studies. Only 20% of clinics included any mention of potential adverse effects associated with psychedelic use. When comparing the most frequently promoted indications on psychedelic clinic websites with the top indications in referenced studies, 2 out of 3 matched, with depression and post-traumatic stress disorder ranking in the top 3. However, within the top 3, cited studies included treatment-resistant depression (TRD) whereas the psychedelic clinics promoted psychedelic use for anxiety.

The reliance on case series or small case-control studies on these websites to promote psychedelics for several different indications raises questions about the credibility of these claims. While some systematic reviews have shown efficacy of psychedelics for certain mental health conditions [12–14], experts have been concerned about the conflict of interest in psychedelic research and the lack of transparency with information sharing with the rise of entrepreneurial interest in this potentially lucrative market [28]. High profile entrepreneurs and corporations have invested significant resources to fund psychedelic research [29]. Many researchers and clinicians are employed as advisors for psychedelic clinics and companies [30]. Interests from large corporations in the psychedelic market in addition to the involvement of researchers in private psychedelic companies should be considered when interpreting the quality of evidence supporting the efficacy of psychedelics for various medical indications. Given that a large, anonymous, online survey has shown that internet websites are the second most common source of information for psychedelics for the public [21], it is important that psychedelic clinic websites use high quality evidence to support their claims. It is integral to explore how knowledgeable providers affiliated with these clinics are on the topic of psychedelic-assisted therapies. An anonymous survey exploring American psychiatrists’ attitudes towards psychedelic-assisted therapies showed that there were limitations in psychedelic knowledge [31]. Psychiatrists desired to learn more about psychedelic-assisted therapies, especially surrounding adverse effects, highlighting the lack of knowledge in this field [31]. Clinicians associated with these clinics have the duty to use appropriate evidence to support their decision making and therapeutic options that are made available to the public.

Most clinics included in our study did not mention potential adverse effects associated with psychedelic use. Although there are studies exploring the relationships between cognitive impairments and prolonged psychedelic use [32–34], these studies tend to be lower quality evidence or may not be clinically translatable or relevant. Historical psychomimetic view, rooted in mid-20^th^ century, framed psychedelic experiences as mimicking psychosis, where the effects of psychedelics were interpreted as insanity or psychotic [35]. In contrast, psychotherapeutic view described psychedelic experiences as mind and spiritual awakening, useful in resolving mental issues in therapeutic settings [35]. Given the potential resemblance between spiritual awakening and psychosis, highly specialized assessment and support is required. It is unclear whether these clinics are equipped with the expertise to identify and manage such significant events should they arise. Collectively, these issues point to the need for more research to better understand the risks and benefits associated with short and long-term psychedelic usage for mental health treatments.

Nine out of ten clinics included in our study offered concurrent psychotherapy, though it remains unclear whether these psychotherapeutic offerings are consistent with the standard therapeutic model published in literature. Standard model generally includes two clinicians per patients, where clinicians have extensive professional experience and training with the effects of psychedelic compound [36]. The clinical team spends a considerable amount of time with patients, approximately 35–40 hours [36]. The “set and setting” play a significant role in shaping the process and outcomes of psychedelic experience. The term “set” encompasses psychological factors such as mood and intentions, while “setting” involves environmental factors such as social and cultural space [35]. Recognizing the importance of these influences, therapeutic alliance and rapport have been described as determinants of efficacy and safety of mental health treatment with psychedelic medicines [36]. A literature review of trials found that adverse events such as mania can be precipitated in patients with bipolar disorder when using psychedelics [37], emphasizing the necessity of well-trained practitioners, and appropriate “set” and “setting” to address these potential adverse effects if they arise. To optimize psychedelic medicine, a structured process of preparation and support during the experience, and integration afterwards is recommended. These steps help shape the mindset and environment of patients for these sessions and provoke reflection afterwards [38]. Clinicians play a crucial role in the safety and efficacy of psychedelic medicine, but clinics do not provide information about the level of training of their practitioners. There are several psychedelic therapy training programs emerging in the space, some funded by industry while others affiliated with academic centres. Guidelines for the practice and certification of psychedelic assisted therapy are needed to support safe and effective use of these substances.

Psychedelic clinics in this study emphasized depression as the leading indication for psychedelic use, while only one clinic promoted their application for TRD. This contrasts with the indications highlighted in cited studies, where depression and TRD were both top indications. There are several treatments available for depression, ranging from psychotherapies to pharmacological therapies [39]. TRD lacks a universally accepted definition, but it is commonly defined as failure to respond to at least two trials of antidepressant pharmacotherapy of adequate dose and duration [40,41]. Although there is overlap in some non-biological and pharmacological treatments of depression and TRD, there are treatments unique to TRD such as somatic or brain stimulation therapies [40]. Furthermore, dosage and duration of these treatments may differ between the two conditions. As a result, some clinics that have claimed indications for depression may rely on evidence primarily related to TRD, which can lead to discrepancies between claimed indications and evidence provided.

Some clinics stated that their services are not covered by Ontario Health Insurance Plan (OHIP), requiring patients to self-fund the expenses. This may result in disparities with access to these treatments. A systematic review has found that there is an association between income inequality and poor mental health [42]. If psychedelic assisted psychotherapy gains wider acceptance and becomes a mainstream component of mental health treatment in Canada, careful consideration is needed regarding whether public coverage is warranted based on safety, efficacy, and efficiency of psychedelic medicine. Considering various social determinants of health and existing health inequities is important to ensure equitable access and outcomes for diverse populations.

## Limitations

We focused exclusively on psychedelic clinics in Ontario, Canada. Although Ontario encompasses 40% of Canada’s population, different provinces may have varying prevalence, policies, and regulations surrounding medicinal psychedelics [43]. We did not independently contact clinics to determine whether a licensed physician or nurse practitioner was involved. Instead, we evaluated whether a psychedelic clinic claimed physician or nurse practitioner involvement on their website as our inclusion criteria. During our assessment, it was observed that many clinics did not specify the name of physician or nurse practitioner involved. Our study used the search terms *psychedelic*, *clinics*, and *Ontario* to identify relevant clinics. This search strategy may have missed clinics that used the specific name of psychedelic, rather than the broad term psychedelic, to advertise their products.

## Conclusion

Psychedelic clinics promote the use of psychedelics for a variety of medical indications using low to moderate quality evidence that are not restricted to peer-reviewed evidence to support their claims. Most clinics omit information regarding their models of care, training of their providers, and the potential negative effects of psychedelics, especially regarding harms associated with spiritual emergence and prolonged usage. Given the high costs of psychedelic assisted therapy and the complex relationship between research and private funding, it is integral to have clear, high-quality evidence widely available to the public and for clinicians and clinics to use appropriate information when communicating with the public.

## Supporting information

S1 Table Characteristics of included studies. (PDF)
