# Supplementary material for: Critical appraisal of evidence supporting prescription of psychedelics from clinic websites in Ontario, Canada
Source: PLoS One. 2024 Oct 24;19(10):e0309911. doi: 10.1371/journal.pone.0309911 (PMC11500855; doi:10.1371/journal.pone.0309911)
Supplement: S1 Table — (PDF) [file pone.0309911.s001.pdf]

S1 Table: Characteristics of included studies

Author	Year	Title	Indication	Type of psychedelic	Study design	Aim	Participants/sample	OCEBM Level of Evidence	Outcomes
Abdallah et al	2022	Dose-related effects of ketamine for antidepressant-resistant symptoms of posttraumatic stress disorder in veterans and active duty military: a double-blind, randomized, placebo-controlled multi-center clinical trial	PTSD	Ketamine	Randomized controlled trial	To test "the efficacy of repeated intravenous ketamine doses to reduce symptoms of posttraumatic stress disorder (PTSD)"	Veterans and service members with PTSD (n = 158) who failed previous antidepressant treatment	2	"The standard ketamine dose ameliorated depression measured by the MADRS significantly more than placebo. Ketamine produced dose-related dissociative and psychotomimetic effects, which returned to baseline within 2 h and were less pronounced with repeated administration. There was no evidence of differential treatment discontinuation by ketamine dose, consistent with good tolerability."
Peili et al	2022	Validation of the Psychological Insight Scale: A new scale to assess psychological insight following a psychedelic experience	Changes in psychological insight after psychedelic experience	Not specified	Survey	To "develop a new scale to measure psychological insight after a psychedelic experience: the Psychological Insight Scale (PIS)."	886 participants recruited "via online advertisements as well as through psychedelic retreat providers"	4	"Principal components analysis of PIS scores revealed a principal component explaining 73.57% of the variance, which displayed high internal consistency at multiple timepoints throughout the study (average Cronbach's α = 0.94)."

Becker et al	2021	Acute Effects of Psilocybin After Escitalopram or Placebo Pretreatment in a Randomized, Double-Blind, Placebo-Controlled, Crossover Study in Healthy Subjects	Depression and anxiety	Psilocybin	Randomized controlled trial	To "investigate the response to psilocybin (25 mg) in healthy subjects after pretreatment with escitalopram or placebo"	Twenty-seven participants were recruited by word of mouth or an advertisement that was posted on the web market platform of the University of Basel	3	"Escitalopram pretreatment had no relevant effect on positive mood effects of psilocybin but significantly reduced bad drug effects, anxiety, adverse cardiovascular effects, and other adverse effects of psilocybin compared with placebo pretreatment"
Gagne et al	2022	Depression, Estrogens, and Neuroinflammation: A Preclinical Review of Ketamine Treatment for Mood Disorders in Women	Mood disorders in women	Ketamine	Review	To "understand how ketamine works in the female brain"	N/A; narrative review	N/A	N/A
Grabski et al	2022	Adjunctive Ketamine With Relapse Prevention–Based Psychological Therapy in the Treatment of Alcohol Use Disorder	Alcohol use disorder	Ketamine	Randomized controlled trial	to investigate "the safety and efficacy of ketamine compared with placebo in increasing abstinence in patients with alcohol use disorder	96 patients with severe alcohol use disorder	2	"There were a significantly greater number of days abstinent from alcohol in the ketamine group compared with the placebo group at 6-month follow-up (mean difference=10.1%, 95% CI=1.1, 19.0), with the greatest reduction in the ketamine plus therapy group compared with the saline plus education group (15.9%, 95% CI=3.8, 28.1)"

Kuc et al	2022	Psychedelic experience dose-dependently modulated by cannabis: results of a prospective online survey	Effects of psychedelics + cannabis	Any of: Psilocybin/magic mushrooms/truffles; LSD/1P-LSD; Ayahuasca; DMT/5-MeO-DMT; Mescaline	Survey	To "explore the subjective effects of psychedelics when used alongside cannabis."	321 people recruited from a "software platform" (https://www.psychedelicsurvey.com/)	4	"The simultaneous use of cannabis together with classic serotonergic psychedelics was associated with more intense psychedelic experience across a range of measures: a linear relationship was found between dose and MEQ, ASC-Vis and EDI scores, while a quadratic relationship was found for CEQ scores. No relationship was found between the dose of cannabis and the EBI"
Davis et al	2021	Ketamine-assisted psychotherapy for trauma-exposed patients in an outpatient setting: A clinical chart review study	Trauma	Ketamine	Retrospective clinical chart review (case series)	To "assess the utility of sublingual ketamine-assisted body-centered psychotherapy in trauma-exposed patients in a real world clinic setting"	18 people "entering ketamine-assisted psychotherapy treatment in an outpatient clinic between 2018 and 2020"	4	"Patients who completed six sessions of ketamine therapy reported meaningful (e.g., medium effect size) improvements in PTSD symptoms (P = 0.058; d = −0.48) and global disability in functioning (P = 0.050; d = −0.52) and statistically significant and meaningful improvements in depression (P = 0.019; d = −0.53). There were no improvements in anxiety symptoms."

Shiroma et al	2020	A randomized, double-blind, active placebo-controlled study of efficacy, safety, and durability of repeated vs single subanesthetic ketamine for treatment-resistant depression	Depression	Ketamine	Randomized controlled trial	To compare "the efficacy and safety of single vs. six repeated ketamine [infusions] using midazolam as active placebo"	54 adults with major depressive disorder "at the Minneapolis Veterans Affairs Medical Center between April 2015 and March 2019.	2	"No significant difference in change of MADRS scores between six ketamine group and single ketamine group at 24 h post-last infusion. Repeated ketamine showed greater antidepressant efficacy compared to midazolam after five infusions before receiving single ketamine infusion. Remission and response favored the six ketamine after infusion 4 and 5, respectively, compared to midazolam before receiving single ketamine infusion... repeated infusions were relatively well tolerated"

Collo and Pich	2018	Ketamine enhances structural plasticity in human dopaminergic neurons: possible relevance for treatment-resistant depression	Treatment resistant depression	Ketamine	Review	To review "recent findings on the mechanism of action of ketamine focusing on mouse mesencephalic and human iPSCs-derived DA neurons"	N/A; narrative review	N/A	N/A
Singh et al	2016	A Double-Blind, Randomized, Placebo-Controlled, Dose-Frequency Study of Intravenous Ketamine in Patients With Treatment-Resistant Depression	Treatment resistant depression	Ketamine	Randomized controlled trial	To evaluate the "efficacy of twice- and thrice-weekly intravenous administration of ketamine in sustaining initial antidepressant effects in patients with treatment-resistant depression"	54 adults with treatment resistant depression recruited from 14 states in the United States between 2012-2013	2	"In the twice-weekly dosing groups, the mean change in MADRS score at day 15 was -18.4 (SD=12.0) for ketamine and -5.7 (SD=10.2) for placebo; in the thrice-weekly groups, it was -17.7 (SD=7.3) for ketamine and -3.1 (SD=5.7) for placebo"

ann het Rot et al	2010	Safety and efficacy of repeated-dose intravenous ketamine for treatment-resistant depression	Treatment resistant depression	Ketamine	Clinical trial/ case series	To test "the tolerability, safety, and efficacy of repeated-dose open-label IV ketamine (six infusions over 12 days)"	"10 medication-free symptomatic patients with TRD who had previously shown a meaningful antidepressan t response to a single dose"	4	"The mean (SD) reduction in MADRS scores after the sixth infusion was 85% (12%). Postketamine, eight of nine patients relapsed, on average, 19 days after the sixth infusion (range 6 days–45 days)."
Zarate et al	2006	A randomized trial of an N-methyl-D-aspartate antagonist in treatment-resistant major depression	Treatment resistant depression	Ketamine	Randomized controlled trial	To "determine whether a rapid antidepressant effect can be achieved with an antagonist at the N-methyl-D-aspartate receptor in subjects with major depression."	Eighteen subjects with DSM-IV major depression (treatment resistant) at the "Mood Disorders Research Unit at the National Institute of Mental Health"	3	Subjects receiving ketamine showed significant improvement in depression compared with subjects receiving placebo within 110 minutes after injection, which remained significant throughout the following week. The effect size for the drug difference was very large (d = 1.46 [95% confidence interval, 0.91-2.01]) after 24 hours and moderate to large (d = 0.68 [95% confidence interval, 0.13-1.23]) after 1 week. Of the 17 subjects treated with ketamine, 71% met response and 29% met remission criteria the day following ketamine infusion.

Dore et al	2019	Ketamine Assisted Psychotherapy (KAP): Patient Demographics, Clinical Data and Outcomes in Three Large Practices Administering Ketamine with Psychotherapy	Treatment resistant depression	Ketamine	Non-randomized trial	"to explore KAP [Ketamine Assisted Psychotherapy] within an analytical framework examining three distinct practices that use similar methods"	"235 patients from three distinct private general psychiatric practices located in Northern California (Wolfson and Dore) and Austin, Texas (Turnipseed) from 2013–2018"	4	" KAP is an effective method for decreasing depression and anxiety in a private practice setting, especially for older patients and those with severe symptom burden"
Greenway et al	2020	Integrating psychotherapy and psychopharmacology: psychedelic-assisted psychotherapy and other combined treatments	Treatment resistant depression	MDMA, Ketamine	Review	To "review the therapeutic mechanisms behind both conventional and psychedelic paradigms, including the evolution of this knowledge and the associated explanatory frameworks"	N/A; narrative review	N/A	N/A
Walsh et al	2021	Ketamine for the treatment of mental health and substance use disorders: comprehensive systematic review	Depression, bipolar disorder, social and generalised anxiety, OCD, PTSD, substance use	Ketamine	Systematic review	To "systematically review the extant evidence on ketamine's effects in treating mental health disorders"	N/A (included 83 publications in systematic review)	1	"Systematic reviews and meta-analyses provide support for robust, rapid and transient antidepressant and anti-suicidal effects of ketamine. Evidence for other indications is less robust, but suggests

			disorders, and Eating disorder						similarly positive and short-lived effects. The conclusions should be interpreted with caution because of the high risk of bias of included studies"
Jones et al	2018	Efficacy of Ketamine in the Treatment of Substance Use Disorders: A Systematic Review	Substance use disorder	Ketamine	Systematic review	To "review the literature on the efficacy of ketamine in the treatment of SUDs [substance use disorders]"	N/A (included 7 publications in systematic review)	1	"Ketamine may facilitate abstinence across multiple substances of abuse and warrants broader investigation in addiction treatment."
McInnes et al	2022	A retrospective analysis of ketamine intravenous therapy for depression in real-world care settings	Depression	Ketamine	"Retrospective analysis"	To "quantify treatment response to KIT in a large sample of patients from community-based practices"	9016 depression patients who received KIT [ketamine intravenous therapy] between 2016 and 2020 at one of 178 community practices across the United States.	4	"53.6% of patients showed a response (≥ 50% reduction in PHQ-9 score) at 14–31 days post-induction and 28.9% remitted (PHQ-9 score drop to < 5). The effect size was d = 1.5. Among patients with baseline suicidal ideation (SI), 73.0% exhibited a reduction in SI. A subset (8.4%) of patients experienced an increase in depressive symptoms after induction while 6.0% of patients reported increased SI"

Feder et al	2021	A Randomized Controlled Trial of Repeated Ketamine Administration for Chronic Posttraumatic Stress Disorder	Chronic PTSD	Ketamine	Randomized controlled trial	To "test the efficacy and safety of repeated intravenous ketamine infusions for the treatment of chronic PTSD"	30 adults with 'chronic PTSD' from outpatient depression program in the United States	2	"The ketamine group showed a significantly greater improvement in CAPS-5 and MADRS total scores than the midazolam group from baseline to week 2. At week 2, the mean CAPS-5 total score was 11.88 points (SE=3.96) lower in the ketamine group than in the midazolam group (d=1.13, 95% CI=0.36, 1.91). Sixty-seven percent of participants in the ketamine group were treatment responders, compared with 20% in the midazolam group."
Hasler	2020	Toward specific ways to combine ketamine and psychotherapy in treating depression	Major depressive disorder, bipolar disorder (depressive episodes)	Ketamine	Narrative review	"outlines some mechanistic hypotheses, how Behavioral Activation, Trauma-Focused Psychotherapies and Humanistic Psychotherapy may specifically prolong ketamine's antidepressant effects."	N/A	5	N/A

Kryst et al	2020	Efficacy of single and repeated administration of ketamine in unipolar and bipolar depression: a meta-analysis of randomized clinical trials	Major depressive disorder / Treatment resistant depression	Ketamine	Systematic review of RCTs	To evaluate the efficacy of single-dose ketamine in different subgroups of patients with major depression and establish whether repeated ketamine administration could be a viable strategy to maintain treatment gains	N/A; 20 publications included in systematic review	1	"The largest effect of ketamine vs. controls in reducing depressive symptoms was observed at 24 h (SMD = − 0.89; 95% CI − 1.24; − 0.53; p < 0.00001); however, a significant difference was shown for up to 7 days after a single dose. Significant differences compared with controls were observed for up to 7 days in treatment-resistant patients and when ketamine was added to ongoing antidepressant treatment, while there were no significant differences at 7 days when ketamine was used as monotherapy."
Marcantoni et al	2020	A systematic review and meta-analysis of the efficacy of intravenous ketamine infusion for treatment resistant depression: January 2009 - January 2019	Treatment resistant depression	Ketamine	Systematic review/ meta-analysis	To "evaluate the effect of intravenous ketamine infusion for patients presenting TRD on depression scores, clinical remission and response rates, and to assess its efficacy over both time and frequency"	N/A; 35 publications included	1	"A strong ketamine effect was observed within 4 hours following a single infusion, and peaked at 24 hours. Ketamine's effectiveness was still present, yet somewhat diminished, 7 days post-infusion. Multiple infusions resulted in an enhanced and prolonged ketamine effect."

Drozdz et al	2022	Ketamine Assisted Psychotherapy: A Systematic Narrative Review of the Literature	Pain, mental health (anxiety) , substance abuse disorders	Ketamine	Systematic narrative review	to "existing evidence regarding present-day practices" [about ketamine assisted psychotherapy]	N/A; 17 publications included	4	N/A
Li et al	2022	Long-term outcomes of repeated ketamine infusions in patients with unipolar and bipolar depression: A naturalistic follow-up study	Uni and bipolar depression	Ketamine	Naturalistic follow-up (case series)	To "examine the long-term outcomes of repeated ketamine infusions in patients with unipolar and bipolar depression"	108 patients "with unipolar and bipolar depression" in China, 2016-2018	4	"Seventy-one (65.7%) of patients completed the 9-month follow-up. On month 9, the response and remission rate were 80.3% and 78.9%, respectively. Among 56 patients who achieved response after the repeated treatment phase, 26 (46.4%) of patients sustained response during 9-month follow-up and their GAF score remained over 70. Sixteen patients relapsed during the 9-month follow-up and 14 (85.7%) of the relapse occurred during the first 2-week follow-up"

Kritzer et al	2022	Ketamine for treatment of mood disorders and suicidality: A narrative review of recent progress	Mood disorders (depression, MDD, TRD, bipolar disorder, anxiety, posttraumatic stress disorder (PTSD)), suicidality	Ketamine	Narrative review	To describe the use of "ketamine for treatment of mood disorders and suicidality"	N/A; narrative review	N/A	N/A
Mandal et al	2019	Efficacy of ketamine therapy in the treatment of depression	Severe depression	Ketamine	Case series	To assess "the effect of subanesthetic dose of ketamine... on depressive and anxiety symptoms"	"Twenty-five drug-free/naïve patients of the male sex, with severe depression having no previous history of psychotic disorder, head injury, organic disorder, cardiological problem, or substance abuse were admitted"	4	"Significant improvement in depression, anxiety, and the severity of illness after 2 weeks and 1 month of the last dose of ketamine. Significant improvement at 1 st h of the first dose was seen in depression and anxiety and not for illness severity. There were transient adverse effects observed in some patients which subsided within 1 h."
Liriano et al	2019	Ketamine as treatment for post-traumatic stress disorder: a review	PTSD	Ketamine	Review	"current literature and theoretical mechanism of action [of ketamine for treatment of	N/A; narrative review	N/A	N/A

						PTSD] is discussed in this manuscript".			
Stein et al	2021	Ketamine for PTSD: Well, Isn't That Special	PTSD	Ketamine	Editorial	Editorial about the use of ketamine for PTSD (Feder et al in this table above)	Editorial	5	N/A
Gorska et al	2019	Magnesium and ketamine in the treatment of depression	Treatment resistant depression	Ketamine	Case series	Examined whether "serum magnesium concentration change over time of ketamine treatment course, also whether association between magnesium concentrations and treatment response measured with Montgomery-Åsberg Depression Rating Scale (MADRS) score occurs"	49 adults with treatment-resistant depression	4	No "correlation between magnesium concentrations and the treatment outcome measures in the course of intravenous ketamine administration in patients with TRD. Also, somatic comorbidities did not impact magnesium levels in the study group.

An et al	2021	Intranasal Ketamine for Depression in Adults: A Systematic Review and Meta-Analysis of Randomized, Double-Blind, Placebo-Controlled Trials	Major depressive disorder / treatment resistant depression	Ketamine	Systematic review	To "assess the efficacy and safety of intranasal ketamine in the treatment of major depressive disorder (MDD), especially treatment-resistant depression (TRD)."	Included 5 RCTs with 858 participants	1	The weighted mean difference of MADRS score was observed to decrease by 6.16 (95% CI 4.44–7.88) in 2–4 h, 9.96 (95% CI 8.97–10.95) in 24 h, and 4.09 (95% CI 2.18–6.00) in 28 day. The pooled relative risk (RR) was 3.55 (95% CI 1.5–8.38, $z = 2.89$, and $p < 0.001$) for clinical remission and 3.22 (95% CI 1.85–5.61, $z = 4.14$, and $p < 0.001$) for clinical response at 24 h, while the pooled RR was 1.7 (95% CI 1.28–2.24, $z = 3.72$, and $p < 0.001$) for clinical remission and 1.48 (95% CI 1.17–1.86, $z = 3.28$, and $p < 0.001$) for clinical response at 28 day.
